# Application of Citrus By-Products in the Production of Active Food Packaging

**DOI:** 10.3390/antiox11040738

**Published:** 2022-04-08

**Authors:** Lourdes Casas Cardoso, Cristina Cejudo Bastante, Casimiro Mantell Serrano, Enrique J. Martínez de la Ossa

**Affiliations:** Chemical Engineering and Food Technology Department, Wine and Agrifood Research Institute (IVAGRO), University of Cadiz, Puerto Real, 11519 Cadiz, Spain; lourdes.casas@uca.es (L.C.C.); casimiro.mantell@uca.es (C.M.S.); enrique.martinezdelaossa@uca.es (E.J.M.d.l.O.)

**Keywords:** citrus waste, supercritical CO_2_, sequential fractionation, supercritical impregnation, antioxidant activity, antimicrobial activity

## Abstract

Some citrus by-products such as orange peel contains valuable compounds that could be recovered and restored into the food chain. In this study, an efficient valorization of orange peel has been investigated using green extraction, fractionation, and impregnation techniques. The first step included its extraction using CO_2_ and ethanol under different pressure (200–400 bar) and temperature (35–55 °C) conditions. The extracts obtained at 300 bar and 45 °C showed strong antioxidant with moderate antimicrobial activity. Then, the extract was subjected to a sequential fractionation process. The fraction obtained at 300 bar, 45 °C, and using 32% ethanol showed the strongest antioxidant and antimicrobial activity with a high extraction yield. Finally, the potential of the two best extracts (obtained at 400 bar and 45 °C before any fractionation and the fractions obtained at 300 bar, 45 °C using 32% ethanol) was determined by conducting an impregnation process to obtain an antioxidant food-grade rigid plastic that would preserve fresh food. The percentage of cosolvent (1 and 2% ethanol), the impregnation time (1 and 3 h), the pressure (200 and 400 bar), and the temperature (35 and 55 °C) were evaluated as variables of this process. The impregnated plastic showed good antioxidant and antimicrobial activities.

## 1. Introduction

Citrus fruits are some of the most abundant and popular crops worldwide. Brazil, China, India, Mexico, Spain, and the USA produce over two-thirds of the world’s citrus fruit crops [1,2]. It is well-known that citrus fruits and their derived products are a rich source of vitamins, minerals, and dietary fibers that are essential for human nutrition, growth, and normal development.

Roughly over 40% of the oranges that are produced globally are processed into an array of commercial products, such as dehydrated citrus products or marmalades, fresh juice, or flavoring agents for other beverages. As a result of this processing, large amounts of waste products, such as after-wash wastewater, solid residues (mainly peels, membranes, and seeds), and semisolid residues, such as the centrifugation pulp that results from the extraction of the juice are disposed of [1,2,3,4]. Consequently, numerous researchers have intended worldwide to develop new processing methods that allow more thorough and efficient exploitation of the different residues resulting from the processing of citrus fruit.

Citrus peel is a waste with large biological value and potential health-promoting benefits [5] because of its content, which includes pectin as well as a number of bioactive compounds such as polyphenols (flavonoids and phenolic acids), carotenoids, and essential oils [6,7,8]. These can be valuable products in the food, pharmaceutical, or cosmetic industries [4,9,10]. Therefore, and aiming for the development of a circular economy, this agricultural waste could be valorized in biorefinery plants while reducing their negative effect on the environment [1].

Different green techniques, such as ultrasonic extraction [11,12], microwave-assisted extraction [13,14], supercritical fluid extraction, or pressurized water extraction, have been used to recover bioactive compounds from citrus peel residues. These technologies have proven to be sustainable alternatives to obtain bioactive compounds from citrus residues as a means to improve the use of these resources [7]. In fact, they have achieved improved extraction yields; less power consumption, shorter processing times as well as other advantages such as minimum or zero use of organic solvents [15,16].

Supercritical fluid extraction is a procedure that combines the use of fluids at moderate temperatures under high pressure with values over their critical point. Supercritical fluids have a high diffusivity and low density, viscosity, and surface tension compared to organic solvents. Carbon dioxide is the most often used supercritical fluid for extraction purposes, since it can operate in a safe and environmentally friendly manner and has hardly any impact on the extracted compounds. Supercritical CO_2_ is generally applied to the extraction of essential oils from citrus fruit residues. According to del Valle et al. [17], the optimal conditions to obtain an orange essential composed of 99.5% limonene using this technique are 125 bar and 35 °C, whereas to extract linalool the conditions changed to 80 bar and 35 °C. If other bioactive compounds, i.e., flavonoids, are to be extracted by means of this technique, an additional cosolvent. with different polarity is to be used, such as ethanol, ethyl acetate, and cyclohexane. Based on their results, ethanol has been proposed as the most adequate modifier [18].

Other authors have recently applied supercritical fluid extraction combined with other extraction methods to fractionate the extract. The addition of polar solvents increases the polarity of the supercritical fluid. Therefore, different cosolvents have been used for the fractionation of tangerine peel [19]. Also, as an example, supercritical CO_2_ (scCO_2_) has been combined with subcritical water for the extraction of mandarin peels [20], or a sequence of scCO_2_ extraction and ultrasound solvent has been applied to the fractionation of the bioactive compounds in orange peel (*Citrus sinensis* L.) [21]. In all these cases, the objective was to obtain fractions with a different composition and bioactivity.

Active food packaging is one of the potential uses for these active extracts with powerful antimicrobial and/or antioxidant properties [22,23]. The active materials interact with the packed food and preserve its nutritional properties while inhibiting the growth of pathogenic as well as non-pathogenic microorganisms. For instance, corn and wheat starch film, combined with different concentrations of lemon essential oil has exhibited both antimicrobial and antioxidant properties [24]. Nevertheless, it has been proven that essential oils undergo an oxidation process that alters the color of bioplastic films developed by the casting method [25]. Oluwasina and Awonyemi (2021) produced bioplastic films from starch and peel-ethanol extract added. The effectiveness of such bioplastic film as a packaging material was studied by wrapping up smoked fish muscle tissue. The results demonstrated that the citrus peel extract can successfully replace essential oil for the production of active packing materials [26].

Numerous techniques can be used to incorporate active compounds into polymer matrices. These include solvent casting [27,28], coating [29], electrospinning or melt extrusion process [30]. Supercritical impregnation has been recently reported as an efficient alternative process that can be used for this purpose [31,32,33]. Thus, scCO_2_ has proven to be an efficient carrier to impregnate bioactive compounds into polymers. This technique is particularly efficient when the active substance presents a high apolarity, since, because of its relatively low critical temperature (31 °C), it exhibits a high solvent and diffusion capacity into different types of matrices.

The application of supercritical techniques to impregnate essential oils into polymers for food preservation has already been reported in the bibliography [34,35,36], but any studies that specifically focus on the impregnation of citrus extracts have been found.

A current trend consists of the production of rigid plastic containers where fresh products can be packed for their direct distribution to consumers. These containers are being widely used in vending machines since they allow the supply of different products. In order to extend the expiration date, such containers would be made of food-grade polymers with preserving properties, which would contribute to the vending of minimally transformed and healthier products. The polymers used for this type of packaging are manufactured by extrusion and thermoforming at temperatures above 200 °C. These high temperatures represent a serious obstacle to the incorporation of active compounds into the polymeric solution that is used at the beginning of the manufacturing process. For that reason, supercritical impregnation represents a feasible alternative that would allow to functionalize this type of packaging material after it has been polymerized and formed. As far as we know, no studies have been published that investigate the impregnation of orange extracts into this type of packaging material. In addition, although the supercritical fractionation and also the impregnation of natural extracts into polymeric matrices have already been analyzed separately, studies that investigate these two processes together are scarce. In this work, the efficient utilization and valorization of bitter orange peel by applying innovative and green extraction, fractionation, and impregnation techniques have been investigated. The first step of this study consists of the extraction of bioactive compounds from orange peel by scCO_2_ using ethanol as cosolvent and under different operating conditions. After that, scCO_2_ together with variable percentages of ethanol as polar cosolvent have been used for the sequential fractionation of the extract in order to obtain bioactive compound-rich fractions. Finally, the efficiency of the process for the impregnation of the bioactive extracts and fractions into food-grade rigid plastics was evaluated.

## 2. Materials and Methods

### 2.1. Chemical Reagents and Raw Materials

Bitter oranges of the variety “naranjo de Sevilla” were harvested from Conil de la Frontera (Andalusia, Spain). The fresh fruits were washed, peeled, and dried in a hot air oven at 50 °C for 24 h. Then, the dried bitter orange peels were ground using a Thermomix crusher provided by Vorwerk (Wuppertal, Germany) and stored at ambient temperature until use.

The polymer used for the impregnation process was food-grade rigid polypropylene (PP). This type of plastic is used for a multitude of purposes, including food and beverage packaging. Its properties according to the manufacturer can be seen in Table 1:

The carbon dioxide (99.99%) was purchased from Abello-Linde S.A. (Barcelona, Spain). The reagents 2,2-diphenyl-1-picrylhydrazyl (DPPH), peptone, sodium chloride, yeast extract, 2,3,5-triphenyl-tetrazolium chloride solution (TTC), barium chloride, sulphuric acid, and the phenolic compound standards were purchased from Sigma-Aldrich (Steinheim, Germany). The *Staphylococcus aureus* (ATCC 6538) strain was purchased from Microbiologics Inc. (St. Cloud, MN, USA).

The HPLC-grade solvents (acetonitrile, ethanol, and formic acid) were supplied by Panreac (Barcelona, Spain).

### 2.2. Extraction and Fractionation at High Pressure

**Figure 1:**
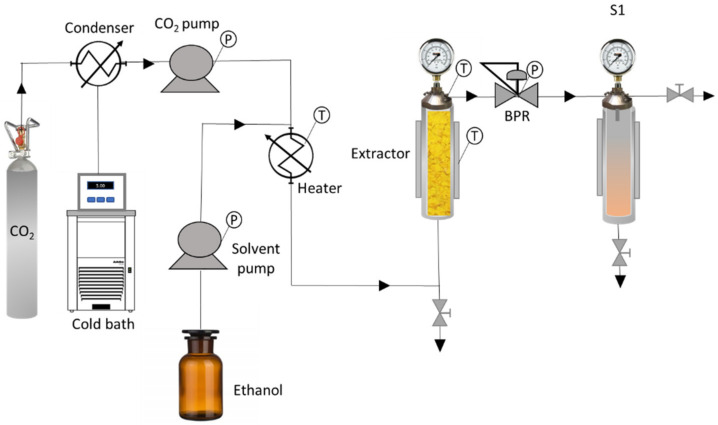
Simplified diagram of the supercritical extraction and fractionation equipment (BPR: automatic back pressure regulator, S1: cyclonic separator, T: temperature, P: pressure).

The equipment used for the extraction experiments was an SF500 model supplied by Thar Technologies (Pittsburgh, PA, USA), fitted with an extractor (500 mL capacity), and with two pumps, both with a maximum flow rate of 50 g/min, one for the CO_2_ and another one for the cosolvent. The pressure was controlled by a Back Pressure Regulator (BPR) and a thermostated jacket was employed to control the extraction temperature. The cyclonic separator allowed the continuous recovery of the extracted material during the process. Figure 1 shows a diagram of the equipment used for this research.

The extraction experiments were carried out at a total flow rate of 25 g/min using CO_2_ + 16% ethanol as cosolvent for 3 h. The effects of the operating conditions, including pressure in the range of 200–400 bar and temperature at 35–55 °C, on the extracts, were evaluated.

**Figure 2:**
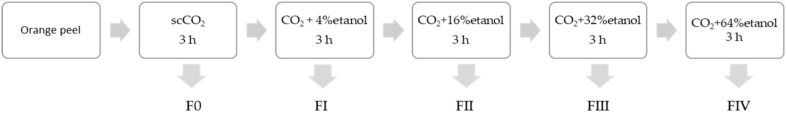
Extraction conditions for sequential fractionation.

All the fractionating experiments were carried out at a flow rate of 25 g/min for 3 h. For the first step, pure scCO_2_ at 300 bar and 45 °C was used as the extraction solvent, and the orange peel residues after the scCO_2_ extraction were used for further extraction of more polar solvents using CO_2_-ethanol mixtures. Previous studies have reported that by raising the percentage of ethanol as a modifier of CO_2_, a significant increment of the phenolic compound yields from citrus peels are achieved [19,20]. Therefore, the range from 4 to 64% ethanol was evaluated. All the experiments were performed at constant 300 bar pressure and 45 °C temperature (Figure 2).

The methodology for the extractions and fractionations involved the loading of the extraction vessel with approximately 144 g of the sample, which had been previously homogenized to present a constant apparent density for all the experiments. The extracts were collected into a cyclonic separator and transferred into glass bottles, which were stored at 4 °C in the absence of light. The experiments were carried out in duplicate for each set of conditions to confirm any variations in the values measured. The total yields obtained were calculated as the total extracted mass divided by the mass of the dry raw material.

### 2.3. Supercritical Impregnation at High Pressure

The impregnation process was carried out using the same high-pressure equipment as described in Section 2.2. Firstly, a specific amount of extract was poured into the vessel. Three plastic samples (9 × 2.5 cm) were maintained in a vertical position within the vessel by placing them on steel supports to avoid any movement of the films during the pressurization and depressurization processes. A four-bladed stirrer (Croschopp, Model PM6015) was incorporated to favor the solubilization of the extract into the scCO_2_. The impregnation processes were carried out in batch mode. The CO_2_ was first pumped at 10 g/min until the desired pressure level was reached. Then, the CO_2_ flow was cut off, and the pressure of the system was maintained throughout the impregnation time. The system was rapidly depressurized (100 bar/min) to produce the impregnated PP.

The plastic samples were subsequently cleaned by means of a wet napkin to remove any extract excess. Each experiment was replicated twice, and the impregnated films were stored at 4 °C to prevent any deterioration prior to their use.

Different experiments were carried out to determine the influence of impregnation time (1 and 3 h), ethanol percentage (1 and 2% of the vessel total volume), pressure (200 and 400 bar), and temperature (35 and 55 °C). The optimal impregnation conditions for each experiment were determined based on the loading of antioxidants that had got impregnated (Equation (2)).

The impregnation process was applied to the two most bioactive extracts. One them obtained without fractionation (400 bar, 45 °C and using the mixture CO_2_ + 16% ethanol) and the second one, was obtained after the sequential fractionation process (FIII: obtained at 300 bar, 45 °C and using 32% ethanol).

### 2.4. Bioactivity of the Extracts and of the Impregnated PP

#### 2.4.1. Determining the Antioxidant Activity by DPPH Assays

Extracts: The antioxidant activity was determined by the DPPH assay [37]. In the presence of the extracts, the DPPH reagent is reduced observing a change in colour from purple to yellow and consequently a decrease in the absorbance measured at 515 nm. Different extracts concentrations in the range of 25 to 1750 µg/mL were prepared in order to determine the efficient concentration (EC_50_) and the inhibition percentage (%*I*). To do so, 0.1 mL of each sample was added to 3.9 mL of 6 × 10^−5^ M DPPH prepared in ethanol. The lecture of the absorbance was done after 2 h of incubation. At that wavelength, the extract did not show any absorbance, so ethanol was used as a blank for the measurements. The percentage of inhibition (%*I*) could be calculated using Equation (1).

(1) $$\%I=\frac{\left(A_{0}-A_{i\;}\right)}{A_{0}}\;*100$$

where *A*_0_ is the initial absorbance and *A_i_* is the final absorbance measured at 515 nm. The assays were done in triplicate.

Impregnated samples: the antioxidant activity of solid matrices was calculated following the method described by Cejudo et al. [33]. A certain amount of impregnated PP was submerged into 4 mL of 6 × 10^−5^ M DPPH solution. In order to let the compound diffuse into the reaction medium, the absorbance was measured at 515 nm after 3 h. Then, the %I was calculated according to Equation (1), calculating the amount of compounds impregnated by Equation (2). The results were expressed as mg antioxidant/100 mg film. The data were done in triplicate.

(2) $$\%I=-0.002C^{2}+0.9042C+4.2439;\;R2^{\;}=0.9989$$

where *C* is the extract concentration.

#### 2.4.2. Antimicrobial Activity

Extracts: the antimicrobial activity against *S. aureus* was determined on the extracts that possess a high antioxidant activity.

The analysis was done in a liquid LB medium using the TTC reagent as an indicator of cell viability. In the presence of viable cells, the reagent turns red, the color of the medium, which can be measured at 490 nm. The analyses were carried out using 96-well microtiter plates following the methods described by Gabrielson et al. [38] and Moussa et al. [39] with modifications. Each well contained 100 µL of 10^6^ CFU/mL bacteria and 10 µL of the extract prepared in the range of 100−20,000 µg/mL. The growth of the bacteria in the presence of the extract was compared with a positive control composed of 100 µL of 10^6^ CFU/mL bacteria and 10 µL of ethanol. Samples were incubated at 37 °C for 24 h. Afterward, 10 µL of 5 mg/mL of the TTC reagent was added to each well. Again, the solutions were incubated for 4 h to allow the reaction of the TTC reagent and the medium. Samples were read using an Epoch 2 spectrophotometer with a microplate reader (Biotek, Winooski, VT, USA). The analyses were done in duplicate, expressing the results as minimal inhibitory concentration (MIC).

Impregnated samples: First, the impregnated polymer samples were sterilized by exposing them to UV light for 15 min. Then, a certain amount was introduced into Pyrex glass tubes (15 × 100 mm) with 10 mL of LB medium. To allow the diffusion of the compounds to the medium, the tubes were incubated at 37 °C for 24 h before inoculation. Then, the absorbance at 625 nm was measured and registered as the initial absorbance (*A*_0_). Afterward, 70 µL of inoculum adjusted to 0.5 McFarland standard was added to each tube achieving a concentration of 1.5 · 10^6^ CFU/mL. The tubes were then incubated for 24 h at 37 °C, when the final absorbance was measured at 625 nm (*A_i_*). All the assays were carried out in triplicate, using as the control sample a tube with LB medium containing a non-impregnated plastic. The results were expressed as the percentage of inhibition (%*I*) by Equation (3).

(3) $$\%I=\left(1-\frac{A_{0}-A_{i}}{A_{control}}\right)*100$$

### 2.5. Analysis of Phenolic Composition

A UPLC separation and identification of the main phenolic compounds were carried out using a Waters Acquity UPLC system (Waters Corps. Milford, MA, USA), equipped with a diode array detector (DAD) and following the method proposed by Shehata et al. (2021) [40] for an Acquity UPLC BEHC18 column with modifications (100 × 2.1 mm with 1.7 μm particle size). The column was set at 47 °C and operated at a flow rate of 0.4 mL/min. The binary system phases were water +0.1% formic acid (solvent A) and acetonitrile +0.1% formic acid (solvent B), with the following phase gradient: initial condition, 95% A for 6 min, 60% A from 6 to 8 min, 10% A from 8 to 10.50 min, 0% A from 11 to 13 min and 95% A from 13 to 14 min. The injection volume was 2.5 μL. The abundance of the main compounds was calculated as the %area in the chromatograms obtained at 280 and 320 nm, depending on their maximum absorption peak. The compounds were identified by comparing the retention times and UV–VIS spectra against those of the commercial standards, attending to the main compounds reported, i.e., naringin (280 nm), luteolin-7-glucoside (320 nm), and hesperidin (320 nm).

### 2.6. Scanning Electron Microscopy (SEM)

The impregnated PP polymers were evaluated by SEM in order to evaluate any possible structural damages after the impregnation process as well as to detect the extract particles on the impregnated PP’s surface. Prior to the experiment, the samples were coated with a thin layer of gold (~15 nm thick) to improve their conductivity. A Quanta 200 scanning electron microscope (Thermo Fischer Scientific, Hillsboro, OR, USA) applying a voltage of 20 kV under vacuum conditions was used.

### 2.7. Statistical Analysis of the Results

To evaluate the influence of the different factors considered on the extraction and impregnation processes the results have been analyzed using the computer application Statgraphics centurion XIX (Corp., Princeton, NJ, USA). The significance levels of the factors have been established at *p* = 0.05 and the sign associated with each factor indicates the positive or negative effect caused by the corresponding variable.

## 3. Results

### 3.1. Extraction of Bioactive Compounds from Orange Peels

**Figure 3:**
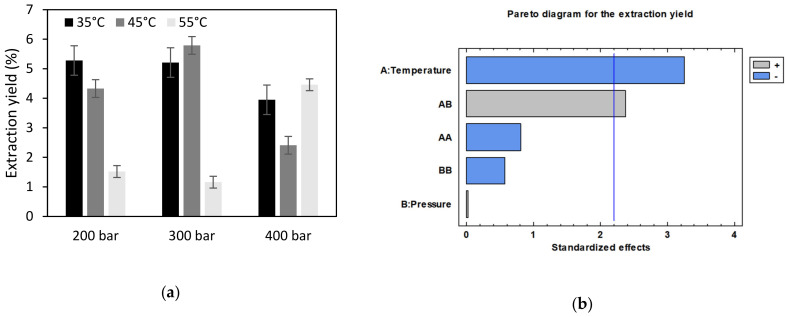

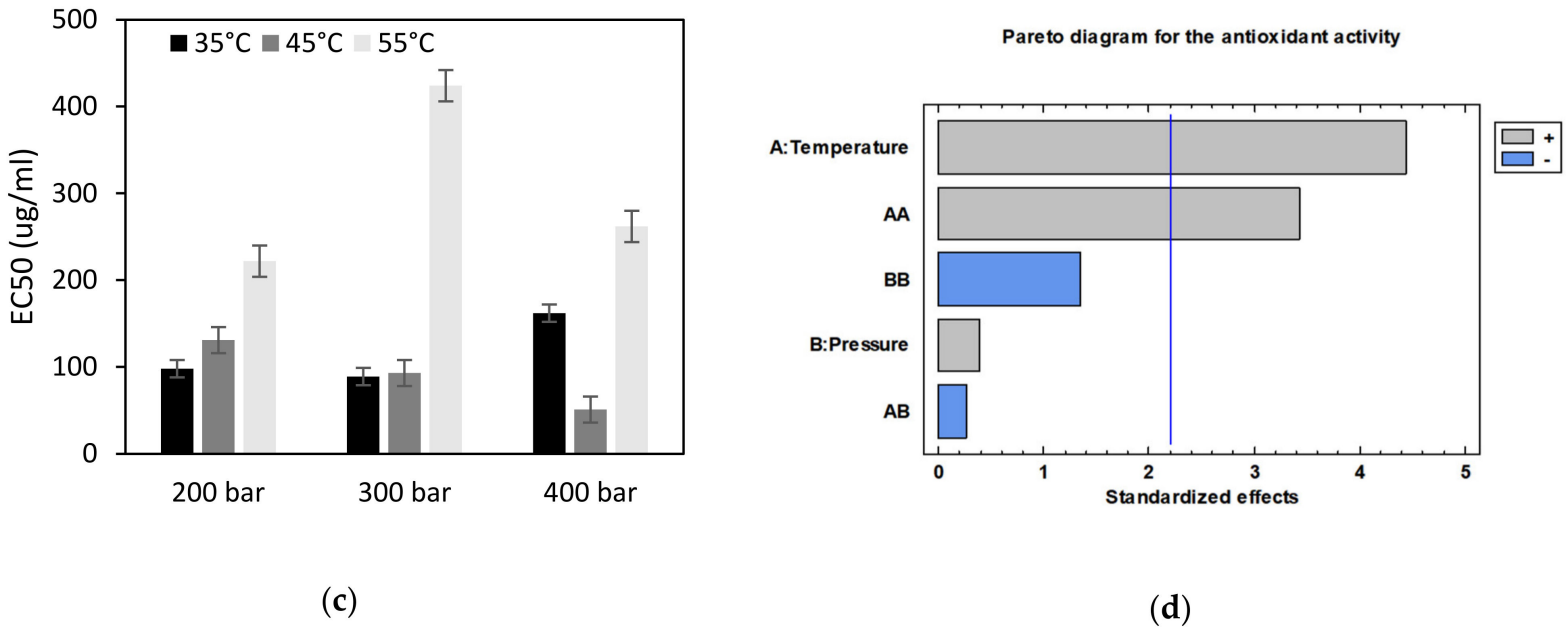
Extraction yield (**a**), Pareto diagram of the extraction yields (**b**), antioxidant activity (**c**), and Pareto diagram of the antioxidant activity (**d**) of the extracts obtained from orange peels using CO_2_ + 16% ethanol (*p* = 0.05).

The extraction yields obtained under different conditions are shown in Figure 3a. Pressure and temperature and, subsequently, CO_2_ density, had some influence on the extraction yields so that the greatest yield was obtained at 300 bar and 45 °C (5.79 ± 0.83%).

The effect of temperature was more evident than that of pressure. Thus, changing the temperature from 35 to 55 °C either at 200 or 300 bar induced a decrease in the extraction yield from 5.24 ± 0.96% down to 1.34 ± 0.76%. At 400 bar, on the other hand, this negative effect of temperature was only observed when it went from 35 °C up to 45 °C. On the contrary, as the temperature was further increased and reached up to 55 °C, an increase in the overall extraction yield was observed. The Pareto diagram in Figure 3b allows to confirm that temperature is the variable with the greatest influence on extraction yields in a negative way. The combination of temperature and pressure also has a significant effect on extract yields, but in this case positively. Thus, at low pressure, high temperatures achieve smaller yields, while under high pressure (400 bar), a higher temperature will result in greater yields.

Orange peels are a rich source of naturally occurring antioxidants. The EC_50_ values obtained varied within a wide range that went from 51 ± 3.2 up to 424 ± 6.2 µg/mL (Figure 3c). Since the antioxidant activity of the extracts largely depends on their composition, it will be affected by extraction conditions. It should be noted that the lower the EC_50_ value, the stronger the antioxidant capacity.

In general, higher temperatures produce extracts with the lowest antioxidant activity. Thus, the extracts obtained at 55 °C at any pressure level registered high EC_50_ values. In fact, the statistical analysis of the design of the experiments shows that the temperature and the quadratic interaction of temperature have a significant influence on the process (Figure 3d). In addition, the yield obtained at 300 bar and 45 °C is the greatest. Generally, the compounds responsible for the antioxidant activity as well as for other bioactivities are phenolic compounds, so it is rather common to find some relation between different bioactivities. Considering the results obtained for antioxidant activity, the antimicrobial activity exhibited by the extracts obtained at 35 and 45 °C will be determined by calculating their Minimum Inhibitory Concentration (MIC) for an important food pathogenic bacteria, *Staphylococcus aureus*. Some differences in the antimicrobial activity of the extracts obtained at different conditions were observed (Table 2). The antimicrobial power of the extracts can be classified according to their MIC [41]. So, the extracts with MIC values lower than 500 µg/mL have a strong antimicrobial capacity, the extracts with MIC values between 600 and 1500 µg/mL have a moderate antimicrobial capacity, and the extracts with MIC values above 1600 µg/mL have a weak antimicrobial capacity. According to this classification, it can be stated that, again, the extract obtained at 45 °C and 400 bar pressure has the strongest bioactivity.

Sometimes, when optimizing an extraction process of any matter in order to obtain larger amounts of bioactive compounds, there could be a loss of selectivity, obtaining also other non-target compounds. Therefore, if a higher bioactivity level of the extract is to be achieved, lower yields maybe sometimes more adequate. In this sense, the fractioning process intends to obtain a fraction that is richer in target compounds. Consequently, a study on the sequential fractionation of the extract obtained at 300 bar and 45 °C was performed in order to compare the results against the most bioactive direct extract, i.e., 400 bar and 45 °C. This comparison should allow us to evaluate whether fractionation is a necessary step to significantly increase the bioactivity of an extract that is already obtained in large yields just by completing the first step.

### 3.2. Sequential Fractionation of the Orange Peel Extract

The fractionation by scCO_2_ and CO_2_-ethanol mixtures in sequential steps is based on the increased polarity of the extraction solvent. This process involves the selective separation of the different compounds according to their saturation in the extraction solvent.

**Figure 4:**
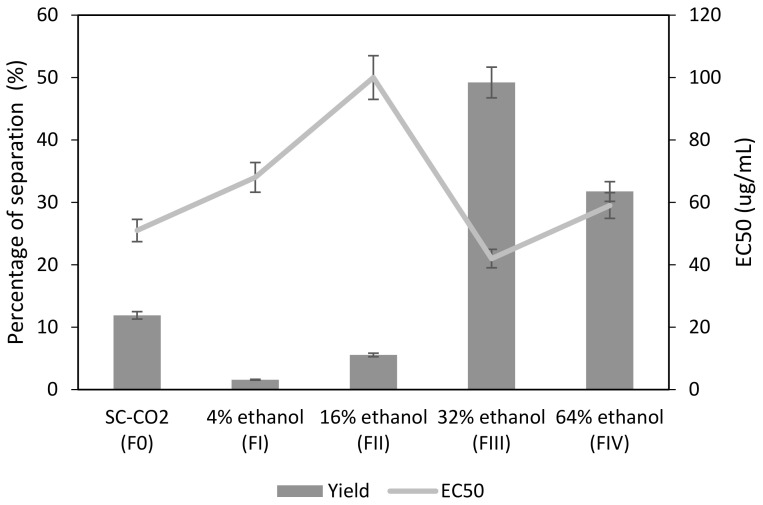
Yields and antioxidant activity of the extracts obtained by sequential fractionation.

Figure 4 shows the percentage of extract obtained under each set of conditions evaluated, where 300 bar and 45 °C have been established as the reference set of conditions. Thus, in the first step pure scCO_2_ was used as the extraction solvent and a large extraction yield was obtained, which was probably formed by the most non-polar compounds, such as the essential oils [17]. The orange peel residue after the first scCO_2_ extraction was subjected to further extraction of other polar components using, in this case, CO_2_-ethanol mixtures. According to the results, a decrease in the extraction yield takes place when 4% ethanol is used. Subsequently, as the amount of ethanol added is increased up to 32%, the extraction yield increases. Finally, when the percentage of ethanol reaches 64%, there is a significant decrease in the extraction yield. With regard to the antioxidant activity of the extracts obtained, the EC_50_ values follow a similar trend to that of the extraction yields. The lowest EC_50_ values, and therefore the highest antioxidant activity, are reached when 32% ethanol is added.

The fraction obtained when 32% ethanol is used as cosolvent exhibited MIC values below 500 µg/mL (50 µg/mL), which would corroborate this fraction as a substance with high antimicrobial properties.

**Figure 5:**
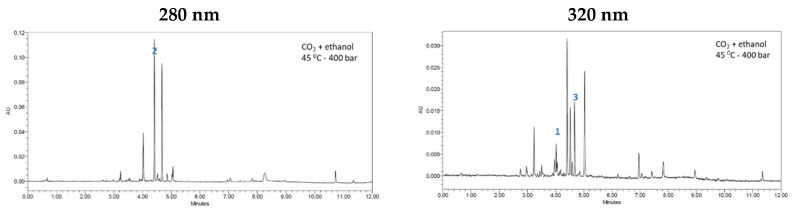

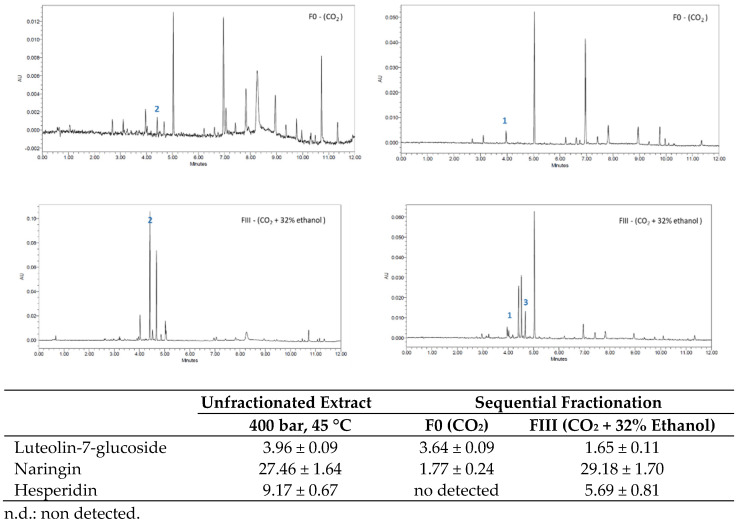
Chromatograms of the extract obtained at 45 °C and 400 bar, and of the fractions F0 and FIII. The table shows the percentage of each compound in the extracts or fractions with 95% confidence intervals.

Figure 5 shows the chromatograms of the extract obtained at 400 bar and 45 °C before any fractionation as well as the fractions F0 and FIII obtained by sequential fractionation. The chromatograms have been measured at either 280 or 320 nm, according to each compound’s maximum absorption region. Three peaks have been identified: (1) Luteolin-7-glucoside; (2) Naringin and (3) Hesperidin. Regarding naringin, a comparison of the chromatograms obtained at 280 nm allow to confirm that the concentration of this compound in the extract obtained without fractionation is slightly lower (27.46 ± 1.64%) than that registered by FIII (29.18 ± 1.70%), which indicates a certain level of purification. The F0 fraction, which was extracted using pure CO_2_, presents very low percentages of this naringin (1.77 ± 0.24%). On the other hand, the percentage of luteolin-7-glucoside is significantly low in FIII, since most of it remains in the F0 fraction, which allows us to infer that the fractionating conditions used are selective to this compound. On the other hand, hesperidin was not detected in the F0 fraction, but FIII presented 5.69 ± 0.81% of this compound. Furthermore, if the chromatograms of the extract obtained at 400 bar and 45 °C are visually compared against the chromatogram corresponding to fraction FIII, it can be observed that the latter shows fewer peaks, which indicates that the compounds are purer.

### 3.3. Impregnation of the Orange Peel Extract and the Purified Fraction

The fraction FIII obtained by sequential fractionation has a strong antioxidant and antimicrobial activity and a large extraction yield (Figure 4). On the other hand, the unfractionated extract, which had been obtained at 400 bar and 45 °C also presents a strong antioxidant and antimicrobial activity, but its extraction yield is moderately lower (2.41 ± 0.78%) (Figure 3, Table 2). Nevertheless, the high level of biological activity of either of these two extracts would justify their use in the impregnation process.

A number of factors regarding the operating conditions of the impregnation process were considered with regard to their influence on the loadings achieved. This included ethanol percentages (1–2%), impregnation time (1 and 3 h), pressure (200 and 400 bar), and temperature (35 and 55 °C). The results have been presented in Table 3.

When the impregnation was carried out using the extract obtained at 400 bar and 45 °C, a significant increase in the extract loading was observed when 3 h impregnation time was used, regardless of the percentage of ethanol used. However, when FIII was used for the impregnation neither the impregnation time nor the percentage of cosolvent seemed to have any influence on the final loading.

**Figure 6:**
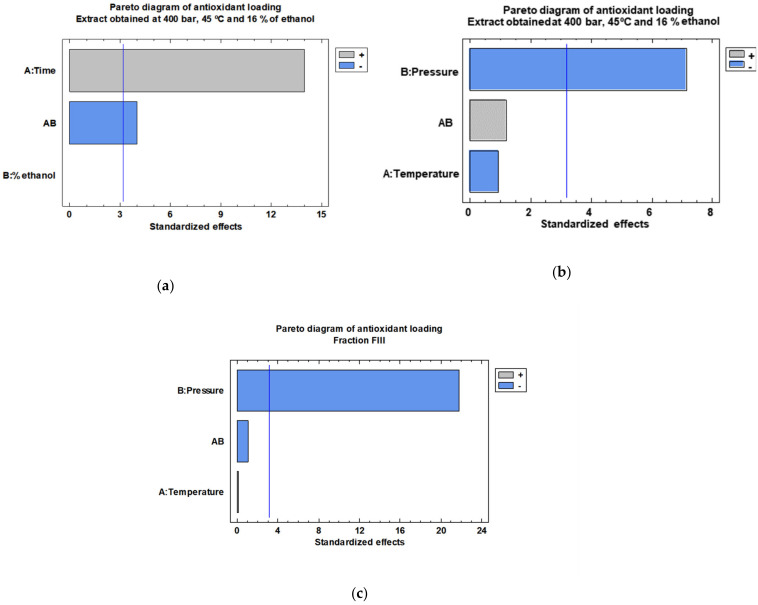
Pareto diagrams of the 400 bar 45 °C extract loading obtained under different impregnation conditions: (**a**) percentage of cosolvent and impregnation time, (**b**) pressure and temperature. Pareto diagram of the FIII fraction loading (**c**) (*p* = 0.05).

The statistical analysis of the experimental design corroborates that when the impregnation is performed using the extract obtained at 400 bar and 45 °C the interaction between impregnation time and impregnation time-percentage of ethanol has a significant effect on the process (Figure 6a). Therefore, a study on the effect of pressure and temperature at a constant 3-h impregnation time and 1% ethanol has been conducted. The influence of pressure or temperature seems to be similar both on extract and fraction on the impregnation processes. In this sense, an isobaric increase in temperature does not have any influence on the extract/fraction loading. However, an isothermal increment of the pressure results in a reduction of the loading in both types of impregnation processes.

**Figure 7:**
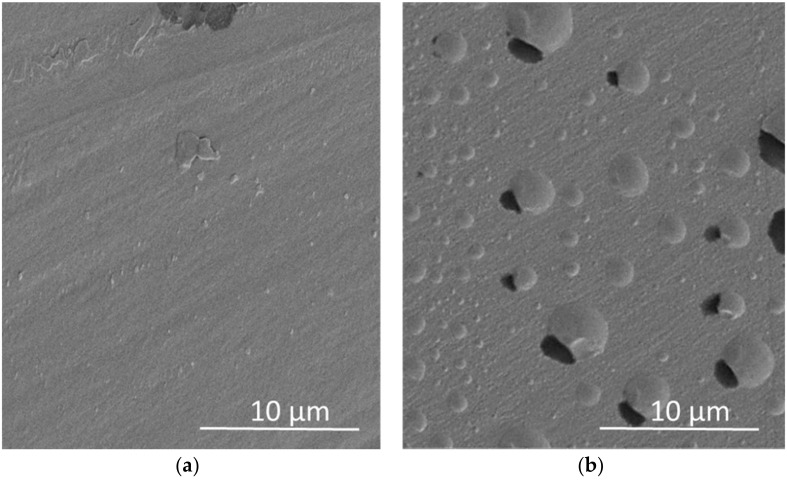
SEM images of the polymer before (**a**) and after (**b**) impregnation.

Despite it was not observed any difference between the appearance of the treated and untreated film to the naked eye, the difference was quite relevant at the microscopical level. Figure 7 displays the surface of the polymer before and after impregnation. The presence of the extract in the impregnated film can be clearly confirmed by a comparison of the two images.

The extract and fraction impregnated polymers obtained at 200 bar, 35 °C, 3 h, and 1% ethanol as cosolvent were evaluated in terms of antimicrobial capacity against *S. aureus*, showing an inhibition of 76.04% ± 4.27 and 69.77% ± 3.25 respectively.

## 4. Discussion

Previous studies on citrus peel extractions (sweet orange, lemon, tangerine, and grapefruit) have used various solvents including water, ethanol, methanol, acetone, petroleum ether, and hexane. This has allowed to confirm that methanol or ethanol was more efficient for the extraction of phytochemicals from citrus peels [40]. The use of ethanol is allowed in the food industry since it is a low toxicity solvent when compared against methanol or other organic solvents. Therefore, ethanol has been the cosolvent chosen to increase the polarity of scCO_2_. Since CO_2_ critical temperature increases when a cosolvent is added to it, subcritical temperatures were employed for the extraction of the bioactive compounds from orange peel. Other advantages associated with the addition of cosolvent to CO_2_ in high proportions are the enhancement of the kinetic desorption of analytes, the inactivation of unwanted enzymes that might destroy certain bioactive compounds, and the reduction of undesired concentration steps [42,43].

With regard to the positive or negative effect of temperature and pressure on the extraction process, the increasing temperature reduces efficiency, while the interaction between pressure and temperature also affects the efficiency of the process and lower yields are obtained (Figure 3b). At low pressure, the effect of temperature as registered in our experiments was similar to that reported by Grosso et al. although they operated within lower pressure (90 and 100 bar) and temperature (40 and 50 °C) ranges [44]. As above explained in the Results section, the temperature has a varying effect on the yield depending on the pressure level. Thus, when the temperature goes up, CO_2_ density is reduced, but at the same time the vapor pressure of the solute increases, and depending on the rest of the operating conditions (mainly pressure), one of the two effects becomes predominant over the other. Thus, at 400 bar and 55 °C, the predominant effect was the increase in the vapor pressure of the solute, thus improving the extraction efficiency. This behavior has been previously described by other authors who worked on similar raw materials [21,34].

The antioxidant and antimicrobial properties of citrus peel depend essentially on the cultivar and may present minor variations depending on fruit ripeness, weather conditions, or other environmental factors. On the other hand, extract quality and composition are strongly dependent on the extraction technique and solvent used. In our case, the extraction operating conditions clearly affected the extracts’ EC_50_ values. The extracts with the highest antioxidant activity, as above mentioned, were obtained at 35 °C and 400 bar. Chen et al. have recently published a paper in which they evaluated the antioxidant activity of different varieties of *Chinese citrus.* The DPPH values of these fruits were, in an ascending order, 295 µg/mL (Kumquat), 785 µg/mL (Mandarin), 900 µg/mL (Lemon), 1555 µg/ mL (Sweet orange), and 2215 µg/mL (Pummelo), respectively. Thus, kumquat displayed the strongest antioxidant capacity, followed by sweet orange, while the weakest antioxidant capacity corresponded to pummelo [45]. The data reported by these authors corroborate those obtained in the present work. In fact, we have produced extracts with even higher antioxidant levels.

Regarding the antimicrobial activity of orange peel extracts, different authors have attributed this activity to the presence of phenolic compounds [41,46]. In general, phenolic compounds exert their antimicrobial activity through various mechanisms. These active compounds can react with microbial cell walls and alter their molecular structure and function or denature certain microbial enzymes. On the other hand, phenolic compounds are complex and include certain nutrients, such as proteins, carbohydrates, minerals, and vitamins, and keep them out of the reach of microorganisms [47,48].

Keeping in mind that the objective of this work is to obtain an extract that can be used to produce active packaging materials, this should exhibit a high antioxidant and antimicrobial activity. Although extracts with the highest antioxidant activity are obtained at 400 bar, the greatest yields were obtained at 300 bar making us select these conditions for the subsequent fractionation study. According to the data in Figure 3 together with those included in Table 2, 300 bar and 45 °C have been established as the most adequate set of conditions to extract active substances from citrus peel, due to its high antioxidant and moderate antimicrobial activity.

According to the data corresponding to the fractionation process (Figure 4), when operating with pure scCO_2_ at 300 bar, 10% of the total extract has provided a fraction that presents a good antioxidant activity. It is well-known that certain compounds such as essential oils, which can be responsible for a high antioxidant activity, can be extracted using scCO_2_ [21,49]. According to the conclusions reached by numerous studies, when essential oils are to be obtained in a particular extract, low pressures seem to be more efficient [18,19]. However, Šafranko et al. studied the scCO_2_ extraction of the volatile compounds in mandarin peel under different pressure levels (100 and 300 bar) and they found that limonene was more abundant when the extraction was carried out at 300 bar [21]. These results are clearly in agreement with those obtained in the present study.

Adding ethanol as a modifier enhances the solvent power of scCO_2_ and promotes the swelling of the matrix. This, in turn, increases the inner volume and the scCO_2_ contact surface [50], which might result in an increased yield. On the contrary, when 4% ethanol is used, the yields are reduced because of the imbibition of the ethanol in the sample. The imbibition time can be usually minimized in supercritical extraction processes by adding the co-solvent to the extractor before the extraction process. Since this method cannot be applied to cascade fractionation processes, it is necessary to increase the extraction time or the working flow rate to counteract the effect of imbibition. It has been observed that extraction yields increase as the amount of cosolvent used is also increased. This is probably explained by the greater solvation power that results from an increased solvent density.

When 64% ethanol was used, a decrease in the yields was registered due to the exhaustion of the raw material. On the other hand, when 32% ethanol was used as cosolvent not only the greatest yields were obtained (Figure 4) and the lowest EC_50_ was registered, but also this fraction exhibited the lowest MIC value (50 µg/mL ± 1.2). Using 32% ethanol as a modifier caused an increment in the antimicrobial activity of the fractions that could be attributed to a more efficient polyphenolic extraction thanks to an increased polarity in the solvent mixture.

Tsitsagi et al. (2018) studied the sequential extraction of tangerine peel to obtain a selective extraction of its bioactive compounds [20]. The first step consisted of the scCO_2_ extraction of essential oil at 100 atm and 35 °C using 15 min equilibrium time. Acetone (7%) was used as a cosolvent in the second step of the extraction to promote the extraction of β-carotene. The optimal parameters were 152 bar, 40 °C, and 1 h equilibrium time, while 1 h was the extraction time for dynamic conditions. Methanol (7%) at 253 bar, 60 °C, 1 h equilibrium time, and 30 min dynamic extraction time were the conditions for the third step of the extraction. Under these conditions, natural flavanones, such as hesperidin, were fractionated.

When it comes to establishing the best conditions to obtain the most suitable extract for the impregnation study, it is necessary to consider both the yields and the activity of the extract obtained. A high yield with a low antioxidant activity may result in a packaging material that would not efficiently preserve food. Accordingly, the best conditions may be different if the extraction process is carried out in a single stage or, otherwise, by fractionation. Thus, in the case of a single-stage extraction process, the best conditions to produce the extract to be used for the impregnation are 400 bar and 45 °C, while in the case of the fractionated extraction, the best conditions would be 300 bar and 45 °C using 32% ethanol as co-solvent. Both substances, i.e., the extract and the fraction, were evaluated for the impregnation of rigid PP plastics. Extract impregnation of polymers is a complex process that depends on many factors. To our knowledge, there are no papers in the literature describing the impregnation of orange peel extracts into food-grade rigid plastics by scCO_2_. It is, therefore, necessary to determine the main factors that may influence the process.

An increment in the loading rate when a cosolvent is incorporated into the supercritical impregnation of different extracts into polymers has been previously reported [51,52]. Therefore, the amount of ethanol to be added is one of the factors that we have determined. Thus, two low percentages (1 and 2%) have been used to ensure that the process takes place within the supercritical range. According to the results obtained, ethanol percentage does not seem to have a relevant influence on the impregnation process when the substance used for the impregnation is the fractionated extract. On the other hand, when the extract that has been produced at 400 bar and 45 °C is used, the interaction between time and ethanol percentage does exhibit a significant influence on the impregnation outcome. Thus, although by increasing the ethanol percentage the solubility of the extracts is increased, at the same time, the affinity between the active substance and the scCO_2_ is also favored. This results in lesser retention of the solute within the matrix [53].

In a previous work, it had been concluded that 1 h is a suitable time to impregnate olive extract in PET/PP films [54]. Based on these results, 1 and 3 h of impregnation time have been tested. When the extract obtained by sequential fractionation was impregnated, time variations did not seem to have any influence on the process, since similar antioxidant loadings were obtained. However, when the extract obtained at 400 bar and 45 °C was the one used for the impregnation, 3 h impregnation time produced larger antioxidant loadings. This could be explained by the fact that the type of compounds that got impregnated into the polymer could be different, as confirmed by the chromatograms in Figure 5.

Although both higher pressure and temperature increased the solubility of the active substance into the scCO_2_, this increment in pressure and temperature also affected the sorption capacity of the polymer. In fact, no larger loadings were achieved when an isobaric increment of the temperature was applied. This could be explained by the fact that both the antioxidant and the non-antioxidant compounds in the extracts compete during the impregnation process not only to dissolve into the supercritical phase but also to reach the active sites in the matrix. Given that a higher temperature improves solubility but affects negatively the sorption power of the matrix, the loadings were always similar regardless of the temperature or the type of active substance used in every case. Similar results were obtained in a study on the impregnation of *Annona muricata* leaf extract into sodium carboxymethyl cellulose [55].

Contrarily, an isothermal increase of pressure caused a decrease in antioxidant loadings. Thus, the increase of the density generates a greater affinity of some of the compounds with the CO_2_. This behaviour favored its desorption during the depressurization stage, which consequently lowered the impregnation yields.

After evaluating the influence of the operating variables on the process, it was necessary to verify that the structure of the polymer was not altered. The small vesicles embedded into the PP correspond to the impregnated extract particles (Figure 7b). The SEM images allowed us to verify that the process to impregnate PP plastic with orange peel extract was efficient since the number of vesicles that could be detected in a small region was quite high. In fact, a highly even distribution of the impregnated extract can be observed. This even diffusion of the extract into the matrix is probably attributable to the good solubility of the extract into CO_2_. This is a quite positive characteristic that makes citrus by-product extracts rather suitable substances to be used for the impregnation of rigid polymers.

## 5. Conclusions

In this paper, scCO_2_ extraction and sequential fractionation have been applied to orange peel to obtain active substances rich in bioactive compounds with potent antioxidant and antimicrobial activities potentially useful in active packaging development. The extracting conditions (mainly temperature) affected qualitatively and quantitatively the extracted compounds and the sequential fractionation of the extract that had been obtained at 300 bar and 45 °C produced fractions with improved bioactivity.

In order to evaluate the suitability of orange peel extracts for the production of food-preserving packaging materials, two of the extracts obtained were selected: the unfractionated extract obtained at 400 bar and 45 °C and the fraction obtained at 300 bar, 45 °C and using 32% ethanol. Four different sets of impregnating conditions have been evaluated and the variables considered were impregnation time, % of ethanol, pressure, and temperature of the scCO_2_. The largest antioxidant loading into the impregnated PP under all the conditions tested was achieved when the extract that had been produced at 400 bar and 45 °C was used. Regarding the evaluation of the antimicrobial activity of the impregnated PP, it was observed that the impregnated PP that had been produced using the fraction provides a stronger inhibition against *S. aureus* (76.04% ± 4.27) than the plastic impregnated with the extract. This could be explained by the type of compounds that had got impregnated into the plastic. Both orange peel supercritical extracts and fractions have proven to be suitable for the production of food-grade plastics with food-preserving properties.

## Figures and Tables

**Table 1:** Properties of the polymer used for the impregnation process.

Molecular formula	-(C_3_H_6_)-n
Semi-crystalline density	0.95 g/cm^3^
Melting point	173 °C
Degradation temperature	287 °C

**Table 2:** Antibacterial activity (MIC) against *Staphylococcus aureus* of the extracts obtained from orange peel under different conditions.

Pressure (bar)	Temperature (°C)	MIC (µg/mL)
200	35	n.d.
300		n.d.
400		1267 ± 4.7
200	45	n.d.
300		806 ± 2.8
400		100 ± 1.1

n.d.: Values greater than1600 µg/mL.

**Table 3:** Extract loadings achieved into PP films under different sets of conditions using unfractionated extract obtained at 400 bar and 45 °C as well as fraction FIII.

Impregnation Conditions	Extract Loading (mg Extract/100 mg Film)	
	Extract (400 bar, 45 °C)	Fraction (FIII) (300 bar, 45 °C)
200 bar-35 °C, 1 h-1% ethanol	0.96 ± 0.12	0.96 ± 0.20
200 bar-35 °C, 1 h-2% ethanol	1.11 ± 0.18	0.93 ± 0.19
200 bar-35 °C, 3 h-1% ethanol	1.56 ± 0.15	0.90 ± 0.12
200 bar-35 °C, 3 h-2% ethanol	1.71 ± 0.12	0.92 ± 0.14
200 bar-55 °C, 3 h-1% ethanol	1.32 ± 0.34	0.93 ± 0.19
400 bar-35 °C, 3 h-1% ethanol	0.67 ± 0.29	0. 51 ± 0.16
400 bar-55 °C, 3 h-1% ethanol	0.75 ± 0.22	0.45 ± 0.15

## Data Availability

All the data presented in this study are available in this article.
